# Association between Nutritional Awareness and Diet Quality: Evidence from the Observation of Cardiovascular Risk Factors in Luxembourg (ORISCAV-LUX) Study

**DOI:** 10.3390/nu7042823

**Published:** 2015-04-14

**Authors:** Ala’a Alkerwi, Nicolas Sauvageot, Leoné Malan, Nitin Shivappa, James R. Hébert

**Affiliations:** 1 Luxembourg Institute of Health L.I.H. (formerly Centre de Recherche Public Santé), Centre d’Etudes en Santé, 1A Rue Thomas Edison, L-1445 Strassen, Grand-Duchy of Luxembourg; E-Mail: nicolas.sauvageot@crp-sante.lu; 2 Hypertension in Africa Research Team (HART), North-West University, Potchefstroom 2520, South Africa; E-Mail: Leone.Malan@nwu.ac.za; 3 Medical Research Council: Research Unit for Hypertension and Cardiovascular Disease, North-West University; Potchefstroom 2520, South Africa; 4 Cancer Prevention and Control Program, University of South Carolina, Columbia, SC 29208, USA; E-Mails: shivappa@email.sc.edu (N.S.); JHEBERT@mailbox.sc.edu (J.R.H.); 5 Department of Epidemiology and Biostatistics, Arnold School of Public Health, University of South Carolina, Columbia SC 29208, USA

**Keywords:** nutritional awareness, diet quality, socio-economic status, dietary energy density, food diversity

## Abstract

This study examined the association between nutritional awareness and diet quality, as indicated by energy density, dietary diversity and adequacy to achieve dietary recommendations, while considering the potentially important role of socioeconomic status (SES). Data were derived from 1351 subjects, aged 18–69 years and enrolled in the ORISCAV-LUX study. Energy density score (EDS), dietary diversity score (DDS) and Recommendation Compliance Index (RCI) were calculated based on data derived from a food frequency questionnaire. Nutritional awareness was defined as self-perception of the importance assigned to eating balanced meals, and classified as high, moderate, or of little importance. Initially, a General Linear Model was fit that adjusted for age, sex, country of birth, and body mass index (BMI). Furthermore, simultaneous contributions to diet quality of individual-level socioeconomic factors, education, and household income were examined across levels of nutritional awareness. Attributing high importance was associated inversely with energy density (*p* = 0.02), positively with both dietary diversity (*p* < 0.0001), and adequacy to dietary recommendations (*p* < 0.0001), independent of demographic factors, weight status and SES. Further adjustment for household income in the EDS-related multivariable model, reduced the β coefficient by 47% for the “moderate importance” category and 36% for the “high importance” category. Likewise, the β coefficient decreased by 13.6% and 10.7% in the DDS-related model, and by 12.5%, and 7.1% in the RCI-related model, respectively, across awareness categories. Nutritional awareness has a direct effect on diet quality, with a minor component of variance explained by improved income. The impact of nutritional awareness on diet quality seems to be a promising area for both health promotion and health policy research.

## 1. Introduction

Despite the remarkable technological progress in health care and treatment, there has been a worldwide increase in lifestyle-related chronic diseases (e.g., type-2 diabetes, cardiovascular disease and obesity) during the last decades [1]. These diseases represent an important cause of premature death and source of prolonged hospitalization, and disability [2]. Cardiovascular disease (CVD) alone accounts for over 4 million deaths yearly, *i.e.* nearly half (49%) of all European mortality, with striking geographical variations [2]. The rise in chronic disease incidence, prevalence and mortality calls into question the effectiveness of existing policies with regard to primary prevention measures, and educational efforts to promote healthy behaviors.

Although the etiology of obesity and chronic diseases is complicated, inappropriate dietary choices, resulting in poor diet quality, is emerging as a major modifiable risk factor [3,4]. Thus far, the determinants of healthy nutrition are poorly understood, but are likely to include individual and environmental factors. In most affluent societies, food availability and easy access to highly processed foods, and sugar-sweetened soft drinks, has been associated with radical transformation of dietary patterns [5]. These, in turn, are assumed to escalating rates of obesity [6].

At the individual level, epidemiologic data have shown that diet quality varies according to age, sex, socioeconomic status (SES), and ethnicity [7,8,9,10]. In addition, nutrition knowledge has a profound influence on food choice and, concomitantly, nutrient intake [11]. Knowledge varies widely across geographical settings [11], which may explain apparent variability in food choices within populations represented by varying cultural backgrounds [12]. The mechanism by which nutrition knowledge transforms into dietary behaviors is intricate. Theoretical models of food choices suggest that individuals’ awareness or tacit assumptions about food are key determinants of food choices [13]. Therefore, self-perception of the importance of balanced meals (hereafter referred to as nutritional awareness) can be viewed as an important factor that may influence dietary choices and nutritional intake [14]. Awareness is modified by knowledge gained through one’s own perceptions or by means of communicated information. In the current study, the term “nutritional awareness” is used to indicate the “perceived importance of dietary balance”.

Against this background, the objectives of the current study are to (1) investigate the independent association between nutritional awareness and diet quality, as indicated by energy density, dietary diversity and adequacy in meeting national recommendations; and (2) elucidate the role of underlying SES factors, measured by means of education and income, on the nutritional awareness-diet quality relationship. Understanding the public’s perception of the importance of balanced meals is essential to assess to what extent the current health promotion messages are put into practice in daily life in order to promote successful healthy eating messages and interventions [14].

## 2. Material and Methods

### 2.1. Study Design and Participants

Analyses were based on data from the Observation of Cardiovascular Risk Factors in Luxembourg (ORISCAV-LUX) survey, a nationwide population-based cross-sectional study of the adult population in Luxembourg. More details of the study design, sample selection, and data collection have been previously reported [15,16,17]. Briefly, a stratified random sample of 1432 participants, aged 18–69 years, was recruited between November 2007 and January 2009 following an invitation letter and phone contact inviting prospective participants to visit the study center. Trained research staff provided the participants with detailed instructions on how to complete the self-administered questionnaire, assisted them in completing the dietary information, and then checked the completeness and accuracy of responses. After data cleaning, particularly for poorly completed Food Frequency Questionnaire (FFQ) [18,19], data from a total of 1351 participants were available for the present analyses.

The present study was conducted according to the guidelines laid down in the Declaration of Helsinki. All procedures involving human subjects were approved by the National Research Ethics Committee (N 200609/03) and the National Commission for Private Data Protection. Written informed consent was obtained from all subjects.

### 2.2. Dietary Intake

The validated semi-quantitative FFQ [18,19] was use to collect data on dietary habits. Participants recorded the frequency of consumption and portion size of 134 foods and beverages.

### 2.3. Outcome Variables: Diet Quality Indicators

Obtaining detailed overall diet quality assessments is challenging in population-based studies. Numerous diet quality indices have been suggested in the literature to reflect various aspects of diet quality [20]. These indices vary from simple tools measuring adherence to dietary recommendations, to complex indices requiring complicated analyses of macro- and micro-nutrient intakes. These indices mainly aim to identify whether different population subgroups are consuming “good/healthy” or “detrimental/unhealthy” foods [20], using a variety of definitions to describe these terms. From among a plethora of such descriptors, we opted to focus on three relevant indicators: energy density [21,22,23], measured by energy density score (EDS); dietary diversity, measured by dietary diversity score (DDS) [24]; and adequacy to achieve national recommendations, measured by recommendation compliance index (RCI) [25], to reflect the overall diet quality [26] and its association with nutritional awareness. Energy density, dietary diversity, and adequacy to dietary recommendations have been identified as key elements of high quality diets [22,27,28].

EDS was calculated as a ratio of total energy intake to daily weight of total food consumed (kcal/g), based on all foods and beverages, excluding drinking water [22]. By choosing the lower energy density option, one can eat a greater volume or weight of an isocaloric food. Therefore, a higher value of EDS indicates more energy per gram of food consumed.

DDS, as described by Kim *et al.* [24], comprised two components: overall variety (daily consumption of at least one serving from each of the five food groups: meat/poultry/fish/egg, dairy products, grains, fruit, and vegetables) (0–15 points), and variety within protein sources (meat/poultry, fish, dairy, beans and eggs) (0–5 points), to yield a total DDS of 20 points (optimal diversity). A diet that has variety within similar food groups, as well as an overall variety, is believed to be superior to a diet with a lower score, indicating more monotonous dietary sources [24]. Variety among the protein sources is included to illustrate the benefits of including diverse sources of food in the diet, even within the same food group [24]. Each item within these food groups provides important nutrient and non-nutrient components (e.g., essential fatty acids from the fish group and phytochemicals from the beans group).

RCI [25] is a composite of 13 food- and nutrient-based components, and ranges between −0.5 (due to a negative half point for excessive salt intake) and 14 points (2 points for high daily fruit and vegetable servings), where a higher degree of adherence results in higher scores. The RCI has been developed to measure the degree of adherence to Luxembourg National Dietary Recommendations, which are consistent with the key prevailing European and international dietary guidelines. The calculation of the DDS and RCI is summarized in Supplementary information; however, detailed information can be found in the respective publications pertaining to each index, as noted above.

### 2.4. Explanatory Variable: Nutritional Awareness

Level of nutritional awareness was assessed by using the question “what importance do you attach to balanced meals in order to feel in a good health?” The answers were classified as: “high importance”, “moderate importance”, and “little importance”. In this study, the term “nutritional awareness” is meant to capture the “participant self-perception of the importance of balanced meals.”

### 2.5. Covariates

Self-reported data on age, sex, country of birth, education, monthly household income, and family size were obtained via a questionnaire. Education was categorized into: “primary”, “secondary”, and “tertiary” level. “Economic status” was ascertained by asking participants to select the category best representing total monthly household income and to indicate the number of adults and children living in the same household, in order to measure the Adult Equivalent Income (AEI). On the basis of the current official national poverty risk threshold for AEI (National Institute of Statistics), the income variable was dichotomized into: “above” or “below poverty threshold”. BMI was computed as weight in kilograms divided by height in metres squared (kg/m^2^).

### 2.6. Statistical Analysis

Descriptive statistics were performed to compare the demographic, socioeconomic and dietary characteristics of participants according to nutritional awareness, using the groups defined above (*i.e.*, of high, moderate and little importance). Percentages (%) were calculated for categorical variables and means and standard deviations (SD) were computed for continuous variables.

General Linear Models (GLM) were computed to examine the association between nutrition awareness and diet quality scores, with adjustment for potential covariates: age in years, sex (male and female), country of birth (Luxembourg, Portugal, other European and non-European countries), BMI (kg/m^2^), and SES, as expressed by level of education (primary, secondary and tertiary) and household income (living above or below poverty threshold).

To explore the potential effect of socioeconomic factors (education and household income) on diet quality across different levels of nutrition awareness, models were fit by adding, successively, education and income. As EDS was based on total daily energy intake, this latter variable was introduced only in the models explaining DDS and RCI.

GLM profile plots were generated to visualize the difference in dietary scores across categories of nutritional awareness, separately according to education level and household income. Results were considered significant at the 5% critical level (*p* < 0.05). All statistical analyses were performed using PASW^®^ for Windows^®^ version 20.0 software, Quarry Bay, Hong Kong (formerly SPSS^®^ Statistics Inc.).

## 3. Results

### 3.1. Characteristics of the ORISCAV-LUX Participants

Overall, more than 50% of the participants attached high importance to eating balanced meals, compared to only 6% who accorded little importance. There were significant differences in the demographic characteristics (age, sex, country of birth), socioeconomic factors (income, but not education level), BMI, and total daily energy intake across the three levels of nutritional awareness. Higher proportions of women and individuals living above poverty threshold were nutritionally aware (attached a higher importance to eating balanced meals). Participants who attached little importance to eating balanced meals were considerably younger and consumed more energy-dense and less diverse foods (Table 1).

### 3.2. Nutrition Awareness and Diet Quality

Independent of demographic factors (age, sex, country of birth), and relative weight status (as expressed by BMI), nutritional awareness (*i.e.*, attributing high importance to eating balanced meals) was inversely associated with energy density of the diet (Model I; *p* < 0.0001) and positively associated with food diversity and adequacy in meeting national recommendations (Model I; both *p* < 0.0001) (Table 2).

Focusing on the relative contribution of each socioeconomic factor separately, the association between nutritional awareness and diet quality scores remained significant across the models. After controlling for education level, there were slight decreases in β coefficients in Model II explaining EDS, and no change observed for DDS and RCI (Table 2).

In contrast, controlling for household income in model III, explaining EDS, resulted in a reduction in the size of β coefficient; by 47% for the category “moderate importance” and by 36% for the category “high importance”. Likewise, adjusting for household income in the model III, explaining DDS, resulted in a reduction of the β coefficient by 13.6% for the category “moderate importance” and of 10.7% for the category “high importance”. Smaller changes were observed in the β coefficients for the models explaining RCI (12.5% for the category “moderate importance” and 7.1% for the category “high importance”) (Table 2).

After controlling for both education and household income (Model IV), the awareness-diet quality association remained significant for EDS (*p* = 0.02), DDS (*p* < 0.0001), and RCI (*p* < 0.0001). These findings indicate that there is an independent association between nutritional awareness and diet quality, regardless of SES; although household income also may partially explain this association (Table 2).

**Table 1:** Description of participants’ characteristics by nutrition awareness, ORISCAV-LUX study, 2007–2008.

Participant’s characteristics	Nutritional Awareness			Total Sample	*p*-value
	High Importance	Moderate Importance	Little Importance		
	*N* (%)	*N* (%)	*N* (%)	*N* (%)	
*n*	700 (51)	570 (42.2)	81(6.0)	1351	
Sex, %					
*Men*	293 (41.9)	310 (54.4)	53 (65.4)	656 (48.6)	<0.0001
*Women*	407 (58.1)	260 (45.6)	28 (34.6)	695 (51.4)	
Country of birth, %					<0.0001
*Luxembourg*	373 (53.3)	391 (68.6)	58 (71.6)	822 (60.8)	
*Portugal*	112 (16.0)	47 (8.2)	3 (3.7)	162 (12.0)	
*Other European*	159 (22.7)	118 (20.7)	15 (18.5)	292 (21.6)	
*Non-European*	56 (8.0)	14 (2.5)	5 (6.2)	75 (5.6)	
Education level, %					0.27
*Primary*	195 (28.3)	133 (23.5)	22 (27.2)	350 (26.2)	
*Secondary*	312 (45.2)	278 (49.1)	42 (51.9)	632 (47.3)	
*Tertiary*	183 (26.5)	155 (27.4)	17 (21.0)	355 (26.6)	
Poverty threshold,					0.006
*Above*	475 (78.4)	405 (80.8)	42 (63.6)	922 (78.6)	
*Below*	131 (21.6)	96 (19.2)	24 (36.4)	251 (21.4)	
	Mean (SD)	Mean (SD)	Mean (SD)		
Age, year	44.9 (13.0)	44.4 (12.8)	38.6 (13.6)	44.23 (13.1)	<0.0001
BMI, kg/m^2^	26.1 (4.7)	27.2 (5.2)	26.5 (5.1)	26.55 (5)	<0.0001
Total energy intake, kcal/day	2322.1 (851.6)	2460 (951.8)	2975 (1255.1)	2419 (935)	<0.0001
Diet quality variables					
Energy density (EDS)	99.1 (26.2)	103.5 (25.8)	115 (35.2)	101.9 (26.9)	<0.0001
Food diversity (DDS)	15.3 (3.2)	14.8 (3.3)	13.4 (3.9)	15.9 (2.6)	<0.0001
Recommendation Compliance Index (RCI)	7.2 (2.3)	6.5 (2.2)	5.3 (2.0)	6.8 (2.3)	<0.0001

*p* Values are from *X*^2^ tests for categorical variables (except for country of birth where Fischer exact test was used), and ANOVA for continuous variables. *p* Values test whether participant’s characteristics vary significantly across categories of nutritional awareness. Mean (SD) indicates mean (standard deviation).

**Table 2:** Regression coefficients and standard error [β and (SE)] for the association between nutrition awareness and diet quality scores, ORISCAV-LUX study, 2007–2008.

Diet quality scores	Nutrition Awareness			*p*-value
	High Importance	Moderate Importance	Little Importance	
	β (SE)	β (SE)	β (SE)	
*Energy density (EDS)*				
Model I	−11.8 (3.1)	−8.3 (3.1)	Ref.	<0.0001
Model II	−11.4 (3.1)	−7.5 (3.1)	Ref.	<0.0001
Model III	−7.6 (3.4)	−4.4 (3.4)	Ref.	0.024
Model IV	−7.4 (3.4)	−3.9 (3.3)	Ref.	0.020
*Dietary diversity (DDS)* *				
Model I	2.8 (0.4)	2.2 (0.4)	Ref.	<0.0001
Model II	2.7 (0.4)	2.2 (0.4)	Ref.	<0.0001
Model III	2.5 (0.4)	1.9 (0.4)	Ref.	<0.0001
Model IV	2.6 (0.4)	2.0 (0.4)	Ref.	<0.0001
Recommendation Compliance Index (RCI) *				
Model I	1.4 (0.3)	0.8 (0.3)	Ref.	<0.0001
Model II	1.4 (0.3)	0.8 (0.3)	Ref.	<0.0001
Model III	1.3 (0.3)	0.7 (0.3)	Ref.	<0.0001
Model IV	1.4 (0.3)	0.8 (0.3)	Ref.	<0.0001

EDS: energy density score, DDS: dietary diversity score; RCI: recommendation compliance index. Ref: reference category. Model I adjusted for age, sex, country of birth, BMI; Model II adjusted to age, sex, country of birth, BMI and education; Model III adjusted for age, sex, country of birth, BMI and income; Model IV adjusted for age, sex, country of birth, BMI, education and income; * All models were additionally adjusted for total daily energy intake. The regression coefficients, β, indicate the difference in diet quality indicators for the categories “high importance” and “moderate importance”, respectively, compared to “little importance”.

**Figure 1:**
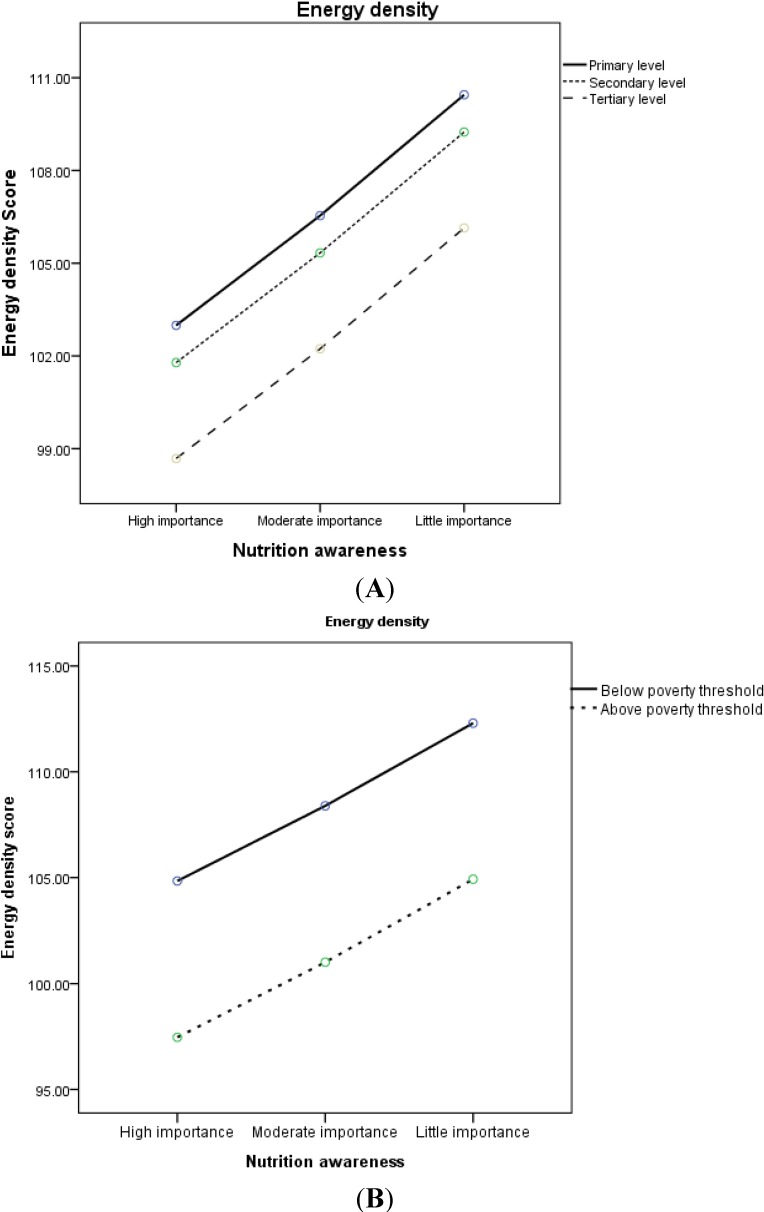
Covariate-adjusted mean * of dietary energy density score across levels of nutritional awareness by (**A**) education level (* adjusted for age, sex, country of birth, BMI, and income); (**B**) household poverty threshold (* adjusted for age, sex, country of birth, BMI, and education level).

Figure 1A, B display the associations between nutrition awareness, as defined in three categories (high importance, moderate importance and little importance) and diet quality indicators (EDS, DDS and RCI), according to education and household income separately. Obviously, the covariate-adjusted mean of energy density increased linearly with decreasing awareness, among participants with primary, secondary and tertiary education. Likewise, there was a linear increase among participants living both below and above poverty threshold.

**Figure 2:**
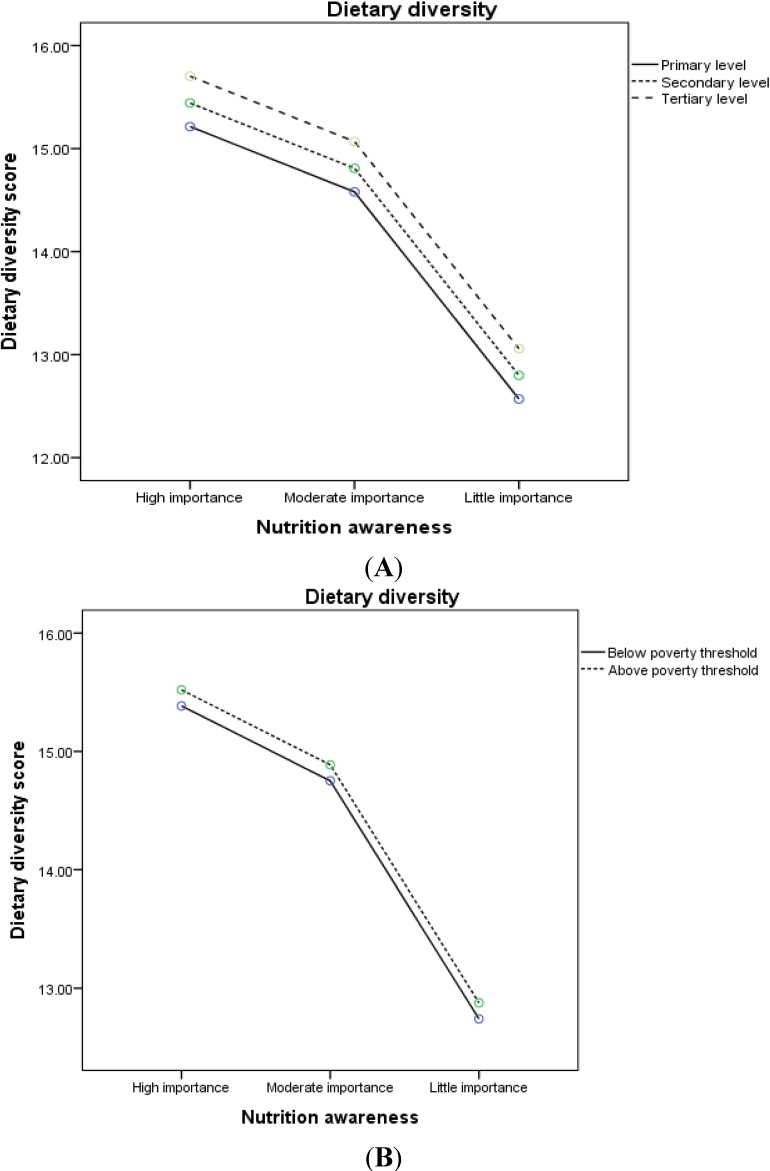
Covariate-adjusted mean * of dietary diversity score across levels of nutritional awareness by (**A**) education level (* adjusted for age, sex, country of birth, BMI, income, and total energy intake); (**B**) household poverty threshold (* adjusted for age, sex, country of birth, BMI, education level, and total energy intake).

Concerning dietary diversity, there was a decline in the covariate-adjusted mean with decreasing nutritional awareness according to education level and household income (Figure 2A, B, respectively). The observed relationship between food diversity and nutritional awareness was non-linear, which means that the adjusted mean of the DDS changed differently by changing the level of nutritional awareness from high to moderate, and from moderate to low. The latter change is marginally larger than the former, suggesting that subjects who attribute high and moderate importance to eating balanced meals select considerably more diverse food compared to those who attribute little importance to diet.

**Figure 3:**
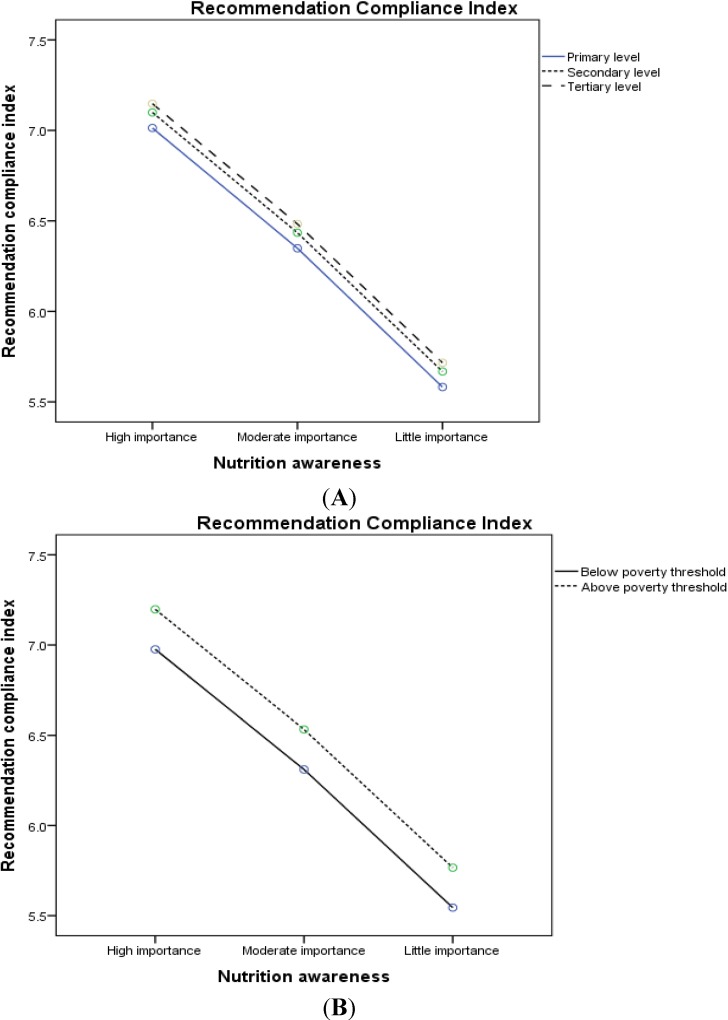
Covariate-adjusted mean * of recommendation compliance index across levels of nutritional awareness by (**A**) education level (* adjusted for age, sex, country of birth, BMI, income, and total energy intake); (**B**) household poverty threshold (* adjusted for age, sex, country of birth, BMI, education level, and total energy intake).

The covariate-adjusted mean of RCI decreased linearly with decreasing nutritional awareness according to both education level and household income (Figure 3A, B, respectively).

## 4. Discussion

This paper provides new evidence concerning the positive relationship between diet quality and perceived importance of dietary balance, as defined by nutritional awareness, and after taking into account socioeconomic factors. These findings further contribute to understanding the nature of such relationships. Important inferences can be derived for health promotion and there are equally important implications for diet- and health-related education policy. Thus, there is potential for these findings to lead to concrete measures to counteract unfavorable trends in poor diet and incidence, prevalence, and mortality due to related chronic diseases.

Nutritionists have long considered adequacy to dietary recommendations, food diversity and low energy-/high nutrient-density as key components of healthy diets [29] and good indicators of diet quality [30]. Diet of good quality is inversely associated with dietary energy density [31], and people eating a variety of foods are more likely to meet their needs for a wide range of essential nutrients [32]. Therefore, most national dietary recommendations strongly promote food diversity along with reducing intake of selected energy-dense foods (*i.e.*, those that are high in fat, refined carbohydrates or both). In this context, the WHO suggests decreasing the consumption of foods that are high in energy density as one approach to help in the prevention of obesity [1].

Interpretation of associations between diet quality and nutritional awareness is complicated by the fact that both are strongly linked to socioeconomic factors. The present study has demonstrated a direct association between perceived importance of dietary balance and diet quality indicators, measured by energy density, dietary diversity and adequacy in meeting dietary recommendations, independent of age, sex, country of birth, BMI and SES.

Our previous research on the ORISCAV-LUX participants provided evidence that national food- and nutrient-related guidelines were not sufficiently appreciated, especially among those attributing little importance to eating balanced meals [25]. The present study confirms that attributing high importance to eating balanced meals was positively associated with the compliance to dietary recommendation, measured by the RCI, independent of their age, sex, BMI, and SES.

Although literature on the nutritional awareness-diet quality relationship is not abundant, our data concur with a French study on middle-aged men, which showed that a better nutritional knowledge is associated with healthy dietary patterns regardless of SES [11]. An American study showed that nutritional knowledge and beliefs modify the association between SES and diet quality [33]. More recently, McLeod *et al.* [34] have reported a mediating role of nutritional knowledge on the socioeconomic gradients in diet quality of Australian first time mothers.

Interestingly, controlling for education level did not change the value of β coefficients in this study, whereas introducing household income in all models of diet quality scores (model III) resulted in attenuation of β coefficients, suggesting that household income exerts a stronger effect than does education on the relationship between perceived importance of eating balanced meals and diet quality. Subjects with greater self-perception of dietary balance and higher incomes tended to be more compliant with dietary recommendations and they had more diverse, and less energy-dense, diets. Possibly, they are more likely to live in environmental conditions that are more conducive to making better food choices [35]; *i.e.*, in the sense that they have better access to good-quality foods. Nutrient-dense diets seem to be preferentially selected by those living above the poverty threshold which also attributes greater importance to healthy dietary habits, in terms of food diversity.

The present results support other studies carried out in children where it was suggested that poorer households consumed less diverse diets compared to wealthier households, and the differences were mainly due to their considerably lower intake of meals containing meat, dairy products and vegetables [32]. A recent systematic review has reported significant, positive associations between greater nutritional knowledge and a higher intake of fruit and vegetables [36]. The price of vegetables and fruit has increased disproportionally over the past 20 years compared to sweets and fats [37]. Increases in food availability and ongoing marketing incentives to increase consumption of low-cost, energy-dense foods may be particularly damaging to the health of lower SES groups, for whom such foods represent a source of more affordable calories [7]. A high energy-density diet is associated with higher disease risk and mortality rates [1]. Darmon *et al.* [38] reported that low-energy-density diets can substantially increase food costs. Therefore, economic resources may be a contributing factor to this nutritional gradient. Additionally, low-cost foods satisfy hunger and are more affordable and more accessible in low-income areas [7].

This study fills a knowledge gap, and enhances the research on socioeconomic disparities in nutrition by addressing nutritional awareness, defined by self-perception of the importance of eating balanced meals in order to maintain good health. Nutritional education efforts *per se* do not necessarily induce dietary change; whereas nutritional awareness as been used here as simple proxy for nutritional knowledge and self-perception of dietary balance, may provide a relevant indicator of willingness and intention to eat to stay healthy. However, further research is needed to suggest a standard definition of “nutritional awareness” that encompasses broader aspects of this term. Other limitations of the current study are its cross-sectional design, which limits making inferences related to causality. Of course, all but prospective studies would be encumbered by this limitation. In addition, an optimal dietary intake assessment strategy still challenges nutrition research [39]. The list of foods in a FFQ is crucially rate-limiting in terms of the ability to capture the variability of dietary habits. This tool has been shown to be sufficiently convenient and inexpensive to use in large-scale, population-based studies [40], although responses rely upon self-report, and therefore are subject to under- and over-reporting biases [41].

The variation in self-perception according to SES and its impact on diet quality could be relevant for public health strategies. The results of the current study suggests that diet quality calls attention to the relationship between perceived importance of dietary balance and consumption of healthy diets, regardless of education level or household income. These results also highlight that income may contribute more to explaining this variation than education does. Health policy makers should take this as a challenge to improve people’s health and dietary status. Most chronic diseases are related to diet. Because diet is related to a host of other health-related behaviors, considering a holistic prevention approach seems worthwhile. In order to increase efficiency of prevention strategies, it might be desirable to focus on less-affluent groups. This departure from “business-as-usual” approach might involve many different stakeholders from a variety of disciplines including decision-makers, food-production industries, teachers, health educators, community activists, and healthcare professionals.

## 5. Conclusions

This study addressed the association between perceived importance of dietary balance and diet quality, while considering socioeconomic status. Most effects of nutritional awareness on diet quality remained significant after adjusting for covariates. Small components of the overall effect of nutrition awareness on diet quality are explained by improved socio-economic status linked to living above the poverty threshold. The impact of perceived importance of dietary balance, as a factor motivating people’s food habits, seems to be a promising area for future health promotion and policy research.

## Supplementary Files

Supplementary File 1
