# Supplementary material for: Association between Nutritional Awareness and Diet Quality: Evidence from the Observation of Cardiovascular Risk Factors in Luxembourg (ORISCAV-LUX) Study
Source: Nutrients. 2015 Apr 14;7(4):2823–38. doi: 10.3390/nu7042823 (PMC4425175; doi:10.3390/nu7042823)
Supplement: Supplementary File 1 [file nutrients-07-02823-s001.docx]

Supplementary Information

**Table S1.** Construct of the Dietary diversity score (DDS) [1].

| Variety | 0–20 points |  |
| --- | --- | --- |
| Overall food group variety (meat/poultry/fish/eggs; dairy/beans; grain; fruit; vegetable) | 0–15 points | ≥1 serving from each food group/day = 15 |
|  |  | Any 1 food group missing/day = 12 |
|  |  | Any 2 food groups missing/day = 9 |
|  |  | Any 3 food groups missing/day = 6 |
|  |  | ≥4 food groups missing/day = 3 |
|  |  | None from any food groups = 0 |
| Within-group variety for protein source (meat, poultry, fish, dairy, beans, eggs) | 0–5 points | ≥3 different sources/day = 5 |
|  |  | 2 different sources/day = 3 |
|  |  | From 1 source/day = 1 |
|  |  | None = 0 |

**Table S2.** Construct of the Recommendation compliance index (RCI) [2].

| **Components** | **National Intake Goals** | **Scoring Criteria** |
| --- | --- | --- |
| **Food-Based Recommendations** | |  |
| Grains products ^a^ | ≥3 times/day | [0–1[ times/day = 0; [1–3[ times/day = 0.5; [3–6[ times/day = 1; ≥6 times/day = 0.5 |
| Dairy products | ≥3 times/day | [0–1[ times/day = 0; [1–2.5[ times/day = 0.5; [2.5–3.5] times/day = 1; >3.5 times/day = 0.5 |
| Meat, poultry, fish and eggs | 1–2 times/day | 0 = 0; ]0–1[ times/day = 0.5; [1–2] times/day = 1; >2 times/day = 0 |
| Sea products | ≥2 times/week | <2 times/week = 0; ≥2 times/week = 1 |
| Fruit and/or vegetables | ≥5 servings/day | [0–3.5[ servings/day = 0, [3.5–5[ servings/day = 0.5; [5–7.5[ servings/day = 1; ≥7.5 servings/day = 2 |
| Salt ^b1^ | 5–10 g/day | >12 g/day = −0.5; ]10–12] g/day = 0; [5–10] g/day = 1; <5 g/day = 1 |
| Non-alcoholic drinks | ≥1.5 L/day | <1L/day = 0; [1–1.5[ L/day = 0.5; ≥1.5 L/day = 1 |
| **Nutrient-Based Recommendations** | |  |
| Total fat ^c^ | ≤35 (%E) | >35 (%E) = 0; ≤35 (%E) = 1 |
| Saturated fatty acid | ≤10 (%E) | >10 (%E) = 0; ≤10 (%E) = 1 |
| Total carbohydrate ^d^ | >45 (%E) | ≤45 (%E) = 0; ]45–75] (%E) = 1; >75 (%E) = 0.5 |
| Simple sugar ^e^ | ≤10 (%E) | >10 (%E) = 0; ≤10 (%E) = 1 |
| Total protein | 15–20 (%E) | <15 (%E) = 0; [15–20] (%E) = 1; >20 (%E) = 0 |
| Total fibers | >25 g/day | ≤25 g/day = 0; >25 g/day = 1 |

^a^ Grains products refer to all types of bread, cereals, muesli, pastries, potatoes, rice, pasta and pulses; ^b1^ This variable doesn’t include the added salt to alimentation; ^b2^ The scoring criteria and cut-off points were based on PNNS-GS with adaptation [3]; ^c^ Refers to the total fat of the diet (oils and fats added and contained in the foods); ^d^ The scoring criteria and cut-off points were defined according to the WHO recommendations [4]; ^e^ The term “simple sugar” refers to all monosaccharides and disaccharides naturally present in foods (honey, syrups and fruit) and added by the manufacturer, cook or consumer. %E Percentage of total daily energy intake. Brackets can be read as follows: “]x” or “x[”: boundary not included, and “[x” and “x]”: boundary included.

References

1. Kim, S.; Haines, P.S.; Siega-Riz, A.M.; Popkin, B.M. The Diet Quality Index-International
   (DQI-I) provides an effective tool for cross-national comparison of diet quality as illustrated by China and the United States. *J. Nutr.* **2003**, *133*, 3476–3484.
2. Alkerwi, A.; Sauvageot, N.; Nau, A.; Lair, M.-L.; Donneau, A.-F.; Donneau, A.-F.; Albert, A.; Guillaume, M. Population compliance with national dietary recommendations and its determinants: Findings from the ORISCAV-LUX study. *Br. J. Nutr.* **2012**, *108*, 2083–2092.
3. Estaquio, C.; Kesse-Guyot, E.; Deschamps, V.; Bertrais, S.; Dauchet, L.; Galan, P.; Hercberg, S.; Castetbon, K. Adherence to the French Programme National Nutrition Sante’ Guideline Score is associated with better nutrient intake and nutritional status. *J. Am. Diet Assoc.* **2009**, *109*,
   1031–1041.
4. World Health Organization. *Diet, nutrition and the prevention of chronic diseases*; WHO: Geneva, Switzerland, 2003.

© 2015 by the authors; licensee MDPI, Basel, Switzerland. This article is an open access article distributed under the terms and conditions of the Creative Commons Attribution license (http://creativecommons.org/licenses/by/4.0/).
